# Prevalence and Determinants of Vaccine ‎Hesitancy Among Students at King Faisal ‎University

**DOI:** 10.7759/cureus.74518

**Published:** 2024-11-26

**Authors:** Ayah Albash, Rana M Alhussain, Norah J Alhajri, Zahra N Alali, Maryam Almulhim, Sayed Ali

**Affiliations:** 1 Medicine and Surgery, King Faisal University, Al-Ahsaa, SAU; 2 Family and Community Medicine, King Faisal University, Al-Ahsaa, SAU

**Keywords:** health, immunity, public health, university students, vaccine hesitancy

## Abstract

Vaccination is a cornerstone of public health, providing immunity against various diseases. However, vaccine hesitancy, as defined by the World Health Organization (WHO), poses a significant challenge to global health efforts. This cross-sectional study explores the prevalence and determinants of vaccine hesitancy among 401 students at King Faisal University. The sample primarily comprises young individuals (97.4% aged 18-24) and predominantly female participants (79%), with 90.5% residing in Al-Ahsaa. Our findings indicate that 75.4% of students adhere to the vaccination schedule, and 55.8% recognize the health benefits of vaccines. Notably, while 1.5% express skepticism, 45.8% strongly agree on the safety of vaccines, and 47.3% acknowledge their effectiveness. Interestingly, only 30.4% feel well-informed about vaccination recommendations, and 47.1% recognize the media's influence on vaccine hesitancy. A significant majority (68.5%) demonstrate high awareness, mainly relying on healthcare professionals (63.4%) for information. Concerns regarding side effects (58.8%) and doubts about vaccine efficacy (21.2%) are prevalent among participants. In conclusion, while the majority of students exhibit high knowledge levels and low hesitancy, concerns about side effects and trust in vaccine efficacy remain critical barriers. Targeted interventions are essential to enhance vaccine uptake and effectively address the factors contributing to vaccine hesitancy.

## Introduction

Vaccine hesitancy is a growing public health challenge that has significant implications for immunization efforts worldwide. The World Health Organization (WHO) defines vaccine hesitancy as the delay or outright refusal of vaccines despite the availability of vaccination services. This behavior can be influenced by a variety of factors, including concerns about vaccine safety, misinformation, cultural beliefs, or a lack of trust in healthcare systems [1,2]. While global vaccination efforts achieve remarkable successes in reducing infectious diseases, the rise of vaccine hesitancy undermines these accomplishments and poses risks to community health.

At King Faisal University in Al-Ahsaa, Saudi Arabia, the student population provides a distinct context for examining the factors contributing to vaccine hesitancy. The university is characterized by a diverse and predominantly young demographic, making it crucial to understand the unique sociocultural influences that shape students’ attitudes toward vaccination. Young adults, particularly university students, are at a critical developmental stage where health behaviors are established, making them a key population for vaccination outreach.

Research indicates that personal experiences significantly shape attitudes toward vaccines, often outweighing empirical evidence [3]. For instance, students may have varying perceptions based on their interactions with healthcare systems or previous vaccination experiences. Negative experiences, particularly those related to perceived side effects, can lead to distrust in vaccines and healthcare providers [4]. In many cases, individuals mistakenly attribute adverse events occurring shortly after vaccination directly to the vaccine, despite these events often being coincidental [5].

Moreover, trust in healthcare providers plays a vital role in influencing vaccine acceptance among university students. A lack of confidence in medical professionals has been strongly correlated with increased vaccine hesitancy [6]. At King Faisal University, many students may rely on informal sources of information such as social media and peer discussions, which often spread misinformation about vaccines. This reliance on non-expert sources complicates their decision-making processes and may reinforce hesitancy [7].

Cultural beliefs and societal norms further contribute to the context of vaccine perceptions at King Faisal University. In Saudi Arabia, cultural factors can significantly influence health behaviors and attitudes toward medical interventions, including vaccination. Understanding these cultural nuances is essential for addressing vaccine hesitancy effectively within this specific population.

This study aims to explore the prevalence and determinants of vaccine hesitancy among students at King Faisal University. By focusing on the sociocultural influences, personal experiences, and trust in healthcare providers, the research seeks to identify specific barriers to vaccine acceptance. The findings will provide valuable insights for public health officials and policymakers, informing targeted interventions that can enhance vaccine uptake among university students and contribute to broader public health objectives.

Rationale of the study

Focusing on college students at King Faisal University is particularly relevant, as this demographic increasingly relies on digital platforms and information technology to guide their health-related decisions- especially during public health crises like the COVID-19 pandemic. Recent research indicates that university students exhibit higher levels of vaccine hesitancy compared to older adults, influenced by factors such as social media misinformation, peer opinions, and individual health beliefs. Preliminary surveys conducted at other universities in Saudi Arabia reveal growing concerns about vaccine hesitancy among students, with many expressing uncertainty about vaccine safety and effectiveness.

These findings underscore the necessity for targeted research at King Faisal University to better understand the specific barriers and fears related to vaccination within this population. The WHO identifies six key determinants of trust - competence, objectivity, fairness, consistency, sincerity, and faith - that can play a crucial role in addressing vaccine hesitancy [8]. By incorporating these determinants into educational materials tailored to the unique context of university students, we can enhance vaccine trust and promote higher vaccination rates.

Effective educational interventions should provide comprehensive information on the benefits and risks of vaccination, specifically addressing students' concerns to mitigate hesitancy. This study aims to identify the fears and barriers contributing to vaccine hesitancy among students at King Faisal University. By doing so, we can inform public health strategies that not only increase vaccination coverage but also enhance community safety and well-being.

Research questions and objectives

This study aims to assess the prevalence and determinants of vaccine hesitancy among students at King Faisal University, Al-Ahsaa, Saudi Arabia. Specifically, it seeks to address the following questions: 1) What is the frequency of vaccine uptake among students at King Faisal University? 2) What barriers do students face in receiving vaccines? 3) What are students' attitudes towards vaccines? 4) How aware are students regarding vaccine safety? 5) What factors contribute to vaccine hesitancy among this population? 6) What are the primary sources of information about vaccines for students?

By answering these questions, the study will provide insights into the levels of vaccine uptake, the nature of hesitancy, and the overall awareness among students, which can inform targeted public health strategies.

## Materials and methods

Study design, setting and duration

A descriptive cross-sectional study was conducted at King Faisal University in Al-Ahsaa, Saudi Arabia from 25 December 2023 to 15 July 2024.

Participants

The study targeted all eligible and accessible students at the university during the study period.

Inclusion and exclusion criteria

The inclusion criteria focused on students currently enrolled at King Faisal University who provided informed consent and completed the survey in full. This ensured that participants were both willing and able to contribute to the study comprehensively. The requirement for complete responses guaranteed that all data collected were robust and minimized gaps in responses, enhancing the reliability of the findings.

Conversely, the exclusion criteria encompassed students not enrolled at King Faisal University, those who declined participation, and those who submitted incomplete surveys. Excluding non-enrolled students maintained the study’s focus on the university population, while excluding incomplete surveys ensured data integrity. By implementing these criteria, the study aimed to capture a representative and complete dataset of eligible participants, allowing for more accurate and meaningful analysis.

Sample size and sampling technique

A total sample of 401 college students was required to estimate an average vaccine hesitancy of 44% with a precision of 5% at a 95% confidence level. The sample size was calculated using the Richard Geiger equation, assuming a design effect of 1. The design effect represented the degree to which the sample size needed adjustment to account for the clustering of responses among participants, ensuring adequate representation of the student population. Participants were recruited through announcements on university platforms, including email newsletters, social media channels, and during university events to maximize reach and accessibility.

Data collection tool and methods

Following ethical committee approval, data were collected using a pre-structured online questionnaire. This questionnaire was developed based on a comprehensive literature review of similar studies and expert consultations. The experts consulted included public health researchers and academic faculty members specializing in vaccination and health behavior, ensuring the questionnaire’s relevance and rigor.

A pilot study involving 30 students was conducted to assess the validity and reliability of the questionnaire. Feedback from this pilot informed necessary revisions to improve clarity and applicability, ensuring that the final questionnaire accurately captured the intended data. The final questionnaire was independently reviewed by two researchers for consistency and accuracy before dissemination.

The study questionnaire was administered online via Google Forms and distributed through social media platforms and university communication channels. The questionnaire comprised two sections: 1. Demographic Data: This section collected socio-demographic information, including age, gender, college, residence, and marital status; 2. Vaccination-Related Attitudes: This section assessed vaccination adherence, students’ attitudes toward vaccines and their safety using a 5-point Likert scale (ranging from strongly disagree to strongly agree), awareness regarding vaccines and their sources of information, and barriers to vaccination.

Statistical analysis

Data were filtered and analyzed using SPSS (IBM Corp., Armonk, NY, USA). The analysis began with descriptive statistics to summarize baseline data. Continuous variables were described using means and standard deviations, while categorical variables were presented as percentages. To evaluate the relationships between variables, categorical variables were analyzed using the chi-square test, and independent t-tests were applied for continuous variables. A confidence interval (CI) of 95% was maintained, with a significance level (p-value) set at ≤0.05. To control for potential confounding variables, multivariate analyses were conducted using logistic regression for categorical outcomes and linear regression for continuous outcomes. This allowed for the assessment of the impact of various demographic and behavioral factors on vaccine hesitancy while adjusting for potential confounders. Variables considered as confounders included age, gender, field of study, and previous vaccination history. The results of the statistical analyses were presented in tables and figures, highlighting key findings and facilitating a clearer understanding of the factors influencing vaccine hesitancy among students at King Faisal University.

Ethical considerations

Ethical approval for this study was obtained from the ethics review committee at King Faisal University in Al-Ahsaa (KFU-REC-2023-DEC-ETHICS1723). The approval process involved submitting a detailed research proposal outlining the study’s objectives, methodology, and ethical implications, along with any relevant documentation, such as consent forms and data handling procedures. Informed consent was obtained from all participants prior to their involvement in the study.

This process involved distributing a consent form alongside the questionnaire. The consent form clearly explained the study's purpose, procedures, potential risks, and benefits, ensuring participants fully understood what participation entailed. Participants were informed that their participation was voluntary and that they could withdraw at any time without any repercussions. To ensure the confidentiality of the anonymously collected data, no personally identifiable information was linked to the survey responses. All data were securely stored in encrypted digital files on password-protected servers and in locked physical locations, accessible only to the research team.

The data were retained for a period consistent with the university’s guidelines before being securely disposed of, ensuring participant privacy was maintained throughout the research process. By implementing these measures, the study aimed to uphold ethical standards and protect the rights and welfare of all participants.

## Results

The demographic breakdown in Table 1 reveals a predominantly young and female sample, with most participants (97.4%) falling within the 18-24 age range and 79% being female. This skew towards younger individuals, likely university students, suggests that the study may have a focus on or appeal to this age group. The residency data indicates that a significant majority (90.5%) are from Al-Ahsaa, reflecting a strong regional concentration. Additionally, most participants are single (89.8%), which is typical for a younger population, with smaller proportions being married (9.7%) or divorced (0.5%).

**Table 1:** Demographic characteristics

Demographic characteristics		N	%
Age	18-24	381	97.40%
	>24	10	2.60%
Sex	Male	82	21.00%
	Female	309	79.00%
Residency	Al-Ahsaa	354	90.50%
	Other	37	9.50%
Marital status	Single	351	89.80%
	Married	38	9.70%
	Divorced	2	0.50%
College	Scientific	245	62.70%
	Humanities	146	37.30%

In terms of academic background, a higher percentage of participants come from scientific colleges (62.7%), while 37.3% are from humanities disciplines. This suggests that the study may have drawn more interest or relevance to those in scientific fields. The gender disparity, with females making up the vast majority, could indicate either a higher level of engagement among women in this particular survey or a reflection of the gender distribution in the sample population. Overall, the table provides a clear picture of a young, largely female, scientifically oriented, and regionally specific sample.

The data from Table 2 highlights a generally positive attitude toward vaccination adherence and health benefits in Saudi Arabia. A significant majority (75.4%) of respondents adhere to the vaccination schedule, reflecting strong compliance with national health guidelines. Additionally, over half (55.8%) strongly believe that vaccinations keep individuals healthy, with another 30.7% agreeing. However, a small minority (1.5%) express skepticism, which may suggest lingering concerns among some about the overall health impact of vaccines.

**Table 2:** Awareness items SA: Strongly Agree, A: Agree, N: Neutral, D: Disagree, SD: Strongly Disagree

Awareness Items		n	%
Do you adhere to the vaccination schedule in Saudi Arabia?	yes	295	75.40%
	no	96	24.60%
Do you think vaccination keeps us healthy?	SA	218	55.80%
	A	120	30.70%
	N	47	12.00%
	D	4	1.00%
	SD	2	0.50%
Do you think vaccinations are safe?	SA	179	45.80%
	A	125	32.00%
	N	75	19.20%
	D	7	1.80%
	SD	5	1.30%
Do you think that all vaccines prescribed in Saudi Arabia are effective?	SA	185	47.30%
	A	136	34.80%
	N	51	13.00%
	D	13	3.30%
	SD	6	1.50%
Do you have sufficient knowledge of the Saudi vaccination program recommendations?	SA	119	30.40%
	A	91	23.30%
	N	101	25.80%
	D	63	16.10%
	SD	17	4.30%
Do you think that frequency of use of 2 and trust in the media are significantly related to vaccine hesitancy?	SA	184	47.10%
	A	127	32.50%
	N	56	14.30%
	D	18	4.60%
	SD	6	1.50%

Regarding the perception of vaccine safety and effectiveness, nearly half (45.8%) of the respondents strongly agree that vaccinations are safe, and 47.3% believe all prescribed vaccines in Saudi Arabia are effective. However, around one-fifth of respondents expressed either neutral or negative opinions on these issues, indicating some level of uncertainty or mistrust. This finding is concerning as public confidence in both vaccine safety and efficacy is critical for achieving high vaccination rates.

Knowledge of vaccination program recommendations appears to be an area of weakness, with only 30.4% strongly agreeing that they have sufficient knowledge. Almost half of the respondents were either neutral or disagreed, showing a significant gap in public awareness. Moreover, 47.1% strongly agree that media influence is tied to vaccine hesitancy, suggesting that improving the accuracy and reach of information disseminated through media channels could play a crucial role in addressing vaccine hesitancy and misinformation.

Table 3 presents the distribution of awareness levels regarding vaccinations, categorized as either "low" or "high." The data indicates that a substantial majority (68.5%) of respondents exhibit a high level of awareness, reflecting a generally well-informed population on vaccination-related issues. This suggests that efforts to disseminate information, whether through public health campaigns or media, have reached a significant portion of the population effectively. Out of the 401 participants, a notable proportion expressed neutral or negative opinions about vaccines. Regarding vaccine safety, 75 respondents (19.2%) were neutral, while seven (1.8%) disagreed and five (1.3%) strongly disagreed, resulting in a total of 87 respondents (21.7%) with neutral or negative views. Similarly, concerning vaccine effectiveness, 51 respondents (13.0%) were neutral, 13 (3.3%) disagreed, and six (1.5%) strongly disagreed, totaling 70 respondents (17.8%) with neutral or negative perceptions.

**Table 3:** awareness levels ‎

Awareness	N	%
Low	123	31.50%
High	268	68.50%

Table 4 presents an analysis of the association between demographic variables (age, sex, residency, marital status, and college type) and awareness levels, with the p-values indicating the statistical significance of these relationships. Although individuals aged 18-24 have a higher proportion of low awareness (32%) compared to those older than 24 (10%), the p-value (0.139) suggests that the difference is not statistically significant. This indicates that age does not have a strong influence on awareness levels in this sample, even though younger individuals show slightly lower awareness. Both sex (p = 0.555) and residency (p = 0.38) show no statistically significant relationship with awareness. Males and females have nearly similar levels of awareness, as do individuals living in Al-Ahsaa compared to those living in other regions. This suggests that gender and geographical location do not play a major role in influencing awareness levels. Although there is a small difference in awareness levels based on marital status, with married individuals having slightly higher awareness (73.7%) than single individuals (68.4%), the p-value (0.089) indicates that this difference is not statistically significant. Likewise, no significant relationship is found between college type (scientific vs. humanities) and awareness (p = 0.489), indicating that educational background in terms of field of study does not strongly affect vaccination awareness.

**Table 4:** Association between demographic variables and awareness The chi-square test was used to assess associations between demographic variables and awareness levels. A p-value ≤0.05 was considered statistically significant. Chi-square values are indicated in the table for each variable comparison.

Demographic Variable	Low Awareness	High Awareness	Total	Chi-Square Value	P-Value
Age (18-24)	122 (32%)	259 (68%)	381	2.18	0.139
Age (>24)	1 (10%)	9 (90%)	10	-	-
Sex (Male)	28 (34.1%)	54 (65.9%)	82	0.35	0.555
Sex (Female)	95 (30.7%)	214 (69.3%)	309	-	-
Residency (Al-Ahsaa)	109 (30.8%)	245 (69.2%)	354	0.77	0.380
Residency (Other)	14 (37.8%)	23 (62.2%)	37	-	-
Marital Status (Single)	111 (31.6%)	240 (68.4%)	351	2.90	0.089
Marital Status (Married)	10 (26.3%)	28 (73.7%)	38	-	-
College (Scientific)	74 (30.2%)	171 (69.8%)	245	0.48	0.489
College (Humanities)	49 (33.6%)	97 (66.4%)	146	-	-

**Figure 1:**
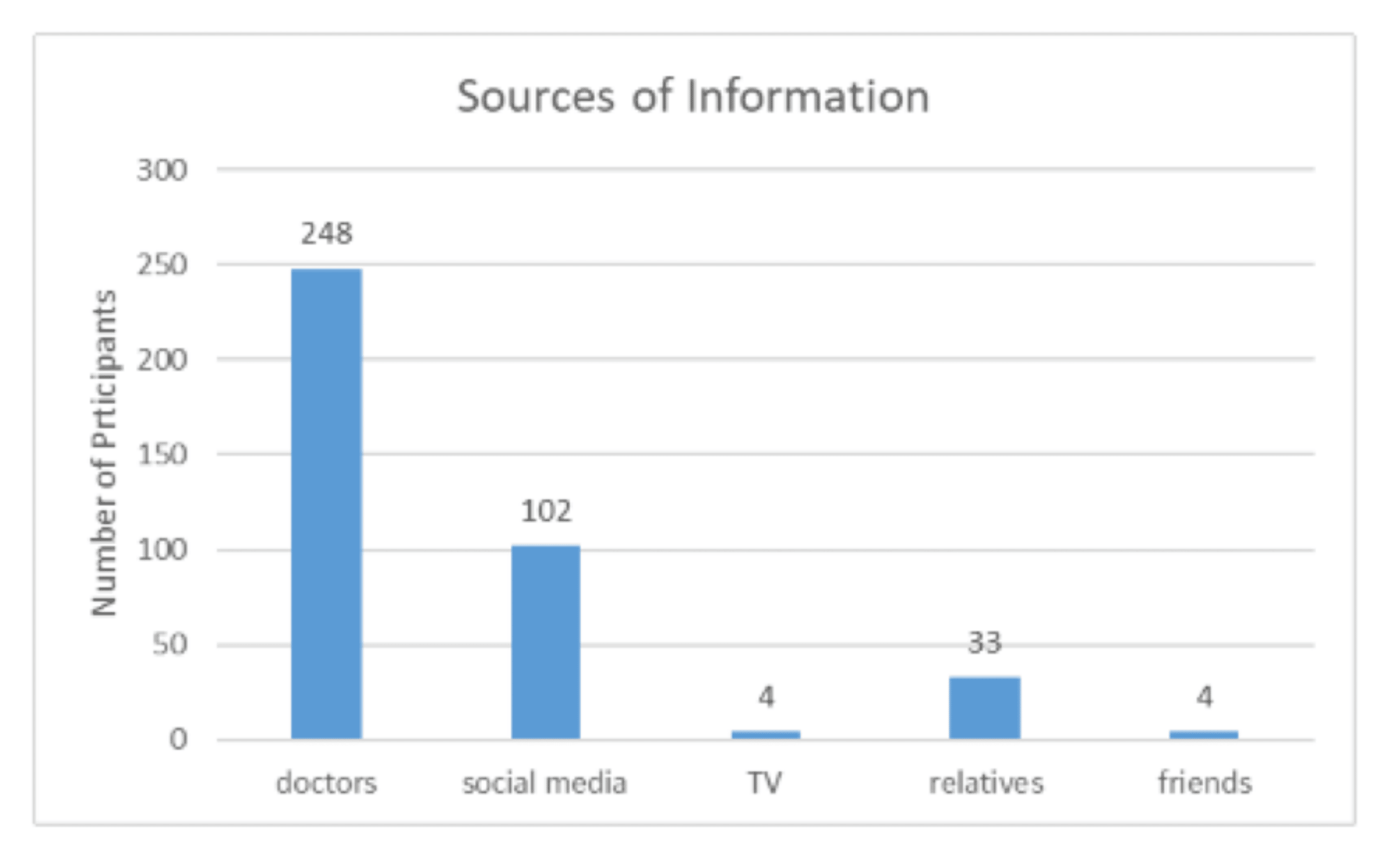
Sources of information

The data provided in Figure 1 shows the primary sources of information about vaccination in Saudi Arabia. The majority of respondents (63.4%) rely on doctors as their main source, indicating that medical professionals are seen as the most trusted and influential figures when it comes to health-related information. This is a positive sign, as accurate information from healthcare providers is critical for fostering understanding and reducing vaccine hesitancy.

Social media is the second most common source, used by 26.1% of respondents. While it offers a wide reach, the content on social media can vary in accuracy, which may contribute to misinformation if not properly regulated or fact-checked.

Other sources such as relatives (8.4%), TV (1%), and friends (1%) play a much smaller role. This suggests that personal networks and traditional media are less significant in shaping public opinion on vaccinations. The low reliance on TV is particularly interesting, given its traditional role in public health campaigns, indicating a possible shift toward digital and direct medical communication in the dissemination of vaccine-related information.

**Figure 2:**
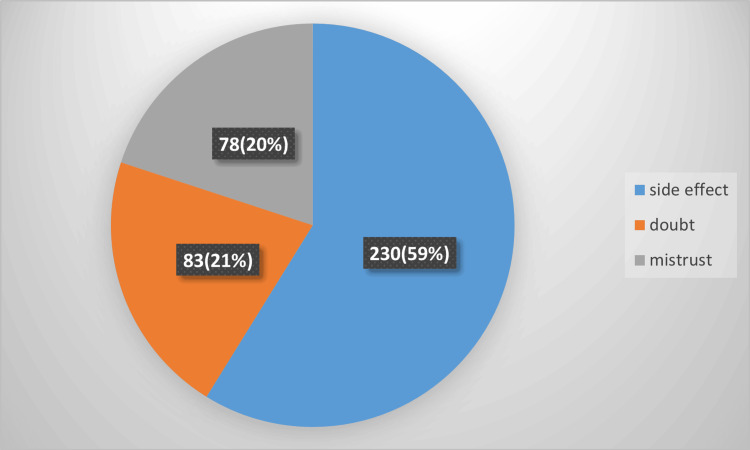
Non-adherence reasons

Figure 2 presents a summary of reported issues related to a specific context, with percentages indicating the proportion of each issue out of the total responses. The most prevalent issue reported is "side effect," which affects over half (58.80%) of the respondents. This suggests that side effects are a significant concern and could indicate a substantial impact on the overall experience or satisfaction. It may be important to further investigate the nature of these side effects and address them to improve outcomes. The next most common issue is "doubt," reported by 21.20% of respondents. This shows that while less common than side effects, doubt is still a notable concern. It may be beneficial to explore the sources of this doubt and work on providing clearer information or reassurance to address these uncertainties. The least reported issue is "mistrust," affecting 19.90% of respondents. Although it is less prevalent than side effects and doubt, mistrust can still affect user satisfaction and engagement. Addressing mistrust through transparency and building confidence in the process or product may be valuable.

## Discussion

The current study aimed to assess the prevalence of vaccine hesitancy and awareness among students at King Faisal University in Al-Ahsaa, Saudi Arabia. Vaccine hesitancy is increasingly recognized as a significant public health concern globally and nationally, as evidenced by its classification as one of the top 10 health risks by the WHO [9]. The study's findings revealed that approximately 75.4% of participants adhered to vaccination schedules, indicating a relatively positive attitude toward vaccination within this population.

The demographic analysis showed a predominantly young (97.4% aged 18-24) and female (79%) sample, consistent with previous research that suggests higher engagement levels among young females in health-related topics. The strong regional representation from Al-Ahsaa (90.5%) further highlighted the localized nature of vaccine perceptions and hesitancy. The findings aligned with Omer et al. [10], who reported that a majority of medical students demonstrated high confidence levels in vaccines and willingness to receive them, mirroring the general optimism about vaccination safety and efficacy observed in our sample.

However, the study also uncovered notable concerns about vaccine hesitancy. While 45.8% of respondents strongly agreed that vaccines are safe, the existence of skepticism (1.5%) and neutral or negative views (approximately 20%) underscored a gap in trust. These findings resonated with Gatwood et al. [11], who noted that about one-third of students exhibited hesitancy toward recommended vaccinations. Notably, the current study identified fear of side effects as the primary concern among over half of the respondents, echoing findings from other studies indicating that fears of adverse events significantly impact vaccination decisions [12].

Additionally, knowledge of vaccination recommendations appeared limited, with only 30.4% feeling well-informed. This suggests a critical area for public health intervention. Similar to results from Nicholls et al. [13], who highlighted that participants were more knowledgeable about the influenza vaccine compared to others, the current study indicates that enhanced communication strategies might be necessary to improve vaccine awareness and mitigate hesitancy. Furthermore, 47.1% of students believed media influence contributed to vaccine hesitancy, pointing to the need for targeted educational campaigns that leverage trusted sources, primarily healthcare professionals, whom 63.4% of participants relied upon for vaccination information.

Interestingly, the analysis of demographic variables showed no statistically significant relationships with vaccination awareness, suggesting that factors influencing vaccine perceptions may transcend typical demographics. This finding is consistent with the notion that vaccine hesitancy is multifaceted, involving personal experiences, societal influences, and broader contextual factors [14,15].

In Saudi Arabia, vaccine compliance is generally high, with a significant majority of the population adhering to recommended vaccination schedules. A cross-sectional study involving 2,030 participants revealed that 90% agreed on the importance of receiving recommended vaccines, 92% believed in their safety, and 91% were committed to administering all recommended doses to their children. Additionally, 86% supported mass or school vaccination campaigns, and 81% were willing to pay for additional vaccines for themselves and their children. These findings indicate a low level of vaccine hesitancy and a positive attitude toward vaccination among the Saudi population.

Limitations of the study

This study has several limitations that should be acknowledged. First, the cross-sectional design restricts the ability to establish causation between vaccine hesitancy and the demographic or behavioral factors assessed. The findings represent associations at a single point in time, which may not capture changes in attitudes over time. Second, the reliance on self-reported data may introduce response biases, as participants may have answered questions in a socially desirable manner rather than reflecting their true beliefs and behaviors. Additionally, the study sample primarily consisted of young adults and predominantly female participants from a single university, which may limit the generalizability of the results to other demographic groups or educational settings. Finally, while efforts were made to ensure a representative sample, potential biases in participant recruitment via university platforms and social media could influence the findings, as students with strong opinions on vaccination may have been more likely to participate. Future research should consider longitudinal designs, more diverse sampling, and potentially objective measures of vaccine hesitancy to address these limitations comprehensively.

## Conclusions

In conclusion, this study revealed that while a majority of students at King Faisal University demonstrate adherence to vaccination schedules and generally view vaccines as safe and beneficial, notable gaps in knowledge and concerns about side effects contribute to some vaccine hesitancy. Trust in healthcare professionals, who serve as primary sources of information, plays a crucial role in shaping positive vaccine attitudes; however, media influence also appears significant, highlighting a need for accurate health communication. To address these issues, targeted educational initiatives that provide clear, reliable information and directly address side effect concerns could enhance vaccine uptake and overall public health outcomes among university students. Further efforts in longitudinal research and broader sampling are recommended to deepen understanding and inform effective interventions.
